# Local and systemic therapy may be safely de-escalated in elderly breast cancer patients in China: A retrospective cohort study

**DOI:** 10.3389/fonc.2022.958116

**Published:** 2022-07-28

**Authors:** Ji Wang, Hongtao Fu, Zhaoyun Zhong, Yunshan Jiang, Hong Pan, Xiaowei Sun, Weiwei Xu, Xinyu Tang, Wenbin Zhou, Shui Wang

**Affiliations:** ^1^ Department of Breast Surgery, The First Affiliated Hospital with Nanjing Medical University, Nanjing, China; ^2^ Jiangsu Key Lab of Cancer Biomarkers, Prevention and Treatment, Jiangsu Collaborative Innovation Center for Cancer Personalized Medicine, School of Public Health, Nanjing Medical University, Nanjing, China

**Keywords:** breast neoplasms, aged, surgical procedures, adjuvant therapy, prognosis

## Abstract

**Background:**

For elderly patients with breast cancer, the treatment strategy is still controversial. In China, preoperative axillary lymph node needle biopsy is not widely used, resulting in many patients receiving axillary lymph node dissection (ALND) directly. Our study aims to determine whether local and systemic therapy can be safely de-escalated in elderly breast cancer.

**Methods:**

Patients aged ≥70 years were retrospectively enrolled from our institution’s medical records between May 2013 and July 2021. Groups were assigned according to local and systemic treatment regimens, and stratified analysis was performed by molecular subtypes. Univariate and multivariate survival analyses were used to compare the effects of different regimens on relapse-free survival (RFS).

**Results:**

A total of 653 patients were enrolled for preliminary data analysis, and 563 patients were screened for survival analysis. The mean follow-up was 19 months (range, 1–82 months). Axillary lymph node metastases were pathologically confirmed in only 2.1% of cN0 cases and up to 97.1% of cN+ cases. In the aspect of breast surgery, RFS showed no significant difference between mastectomy and BCS group (p = 0.3078). As for axillary surgery, patients in the ALND group showed significantly better RFS than those in the sentinel lymph node biopsy (SLNB) group among pN0 patients (p = 0.0128). Among these cases, the proportion of cN+ in ALND was significantly higher than that in SLNB (6.4% vs. 0.4%, p = 0.002), which meant axillary lymph nodes (ALNs) of ALND patients were larger in imaging and more likely to be misdiagnosed as metastatic. With regard to adjuvant therapy, univariate and multivariate analyses showed that RFS in different comprehensive adjuvant regimens were similar especially among hormone receptor (HR)+/human epidermal growth factor receptor 2 (HER2)− subgroup where patients who did not receive any adjuvant therapy accounted for 15.7% (p > 0.05).

**Conclusions:**

It is feasible to reduce some unnecessary local or systemic treatments for elderly breast cancer patients, especially in HR+/HER2− subtype. Multiple patient-related factors should be considered when making treatment plans.

## Introduction

Breast cancer is one of the most common malignant tumors in women, and its incidence is gradually increasing, seriously endangering women’s health (1). In recent years, comprehensive therapy including surgery, chemotherapy, radiotherapy, molecular targeted therapy, and endocrine therapy has become the standard treatment for breast cancer, which has greatly improved the survival of breast cancer patients (2). In the diagnosis and treatment of breast cancer, elderly patients cannot be ignored because their physiological and social characteristics are very different from those of young patients. A study based on the Surveillance, Epidemiology, and End Results (SEER) database showed that breast cancer patients aged 65 or older accounted for nearly half of all newly diagnosed breast cancer patients (3).

Elderly breast cancer is usually larger in size and higher in clinical stage at initial diagnosis. However, compared with young patients, elderly breast cancer has a higher degree of differentiation and more favorite biological behavior. In this population, the expression level of estrogen receptor (ER) and progesterone receptor (PR) is high, and the overexpression rate of human epidermal growth factor receptor 2 (HER2) is low. In addition, early axillary lymph node (ALN) metastasis is less frequent in elderly patients (4–7). At present, the treatment strategy of elderly breast cancer is still based on the reference of young patients, and there are no specific diagnostic and treatment standards for elderly patients. The process of breast cancer treatment often brings some side effects and complications, such as lymphedema, upper limb disorder, fatigue, osteoporosis, and cancer treatment-related cardiac dysfunction (8–11). Besides, comorbidity is present in many elderly breast cancer patients, such as hypertension, coronary heart disease, diabetes, and cerebrovascular diseases. These patients may be intolerant to the toxicity of chemotherapy and radiotherapy. Therefore, personalized treatment for this part of the population is particularly important in clinical practice.

In China, preoperative axillary lymph node needle biopsy is not widely used, resulting in many patients receiving directly axillary lymph node dissection (ALND) according to positive findings in clinical or imaging assessment of ALNs. A number of studies have been carried out to compare the efficacy and prognosis at one or two points in local and systemic treatment of elderly breast cancer patients (12–15). In China, nevertheless, few studies have comprehensively and systematically analyzed the effect of local and systemic therapy on prognosis based on molecular subtypes. Our study aims to discuss whether local and systemic therapy can be safely de-escalated in elderly breast cancer patients aged 70 years or older. Meanwhile, by analyzing the relationship between pathological and preoperative ALN status, we investigated the non-necessity of excessive axillary surgery and the vital role of accurate preoperative ALN assessment in a subset of elderly patients.

## Patients and methods

### Study design and participants

Patients were retrospectively enrolled based on the medical records of our institution’s clinical diagnosis and treatment system between May 2013 and July 2021. Inclusion criteria were as follows: invasive breast cancer, 70 years or older at diagnosis, stage I–III, and clinicopathological data available. Exclusion criteria were as follows: male breast cancer, carcinoma *in situ*, patients with distant metastases, patients who received neoadjuvant therapy of any regimen before surgery, and data on clinicopathological characteristics, surgery, and adjuvant therapy completely missing. This study was approved by the Institutional Review Boards of the First Affiliated Hospital with Nanjing Medical University.

### Patient-related data

Data related to age at diagnosis, clinical stage, breast surgery, axillary surgery, pathological type, size of invasive carcinoma, pathological ALN status, ER, PR, HER2, Ki-67, chemotherapy, radiotherapy, and endocrine therapy were obtained from medical records of our institution. A score of ER or PR ≥1% in immunohistochemical (IHC) staining was considered ER or PR positive. ER+ and/or PR+ were collectively referred to as hormone receptor (HR) positive. HER-2+ was defined as an IHC score of 3+ or an IHC score of 2+ and gene amplification in fluorescence *in situ* hybridization (FISH). Patients were classified as HR+/HER2−, HER2+, and triple negative breast cancer (TNBC) based on IHC and/or FISH results (molecular subtypes). The staging of tumor followed the American Joint Committee on Cancer (AJCC) staging system (8th edition) according to original tumor clinicopathological data. The chemotherapy regimens involved in this study were mainly based on taxanes (such as paclitaxel and docetaxel) and/or anthracyclines (such as doxorubicin and epirubicin), while endocrine regimens were mainly based on tamoxifen (or toremifene), ovarian function inhibitors, and aromatase inhibitors. However, concrete protocol, dose and duration of chemotherapy, radiotherapy, and endocrine therapy were hard to obtain and confirm. Hence, variables involving adjuvant therapy were recorded as yes or no. Data on anti-HER2 therapy were not included in the analysis due to the time span of our study and some missing data on molecular targeted therapies.

### Follow-up and study outcomes

Patients’ survival data were obtained mainly through telephone or outpatient follow-up. The primary outcome of our study was relapse-free survival (RFS), which was defined as months from surgery date to local, regional, or distant metastases, second primary breast cancer, contralateral breast cancer, and death from any cause. If the patient was lost to follow-up, her last outpatient visit information will be reviewed. Due to the low incidence of death events, overall survival (OS) and breast-cancer-specific survival (BCSS) were not analyzed in the study statistics.

### Statistical analysis

Patient ages were represented by means and ranges. The correlations of other categorical variables were analyzed by Fisher’s exact test. Student’s t-test or analysis of variance (ANOVA) was used to compare differences in continuous variables. In the univariate survival analysis, the Kaplan–Meier method was applied to draw survival curves, and log-rank test was used to compare differences in survival between groups. In multivariate survival analysis, Cox proportional hazards model was used to calculate hazard ratios and 95% confidence intervals (CIs). In univariate and multivariate survival analyses, cases with any missing data on included factors were excluded. p < 0.05 was considered statistically significant. Data were analyzed *via* Stata version 16.0.

## Results

### Patients

**Figure 1:**
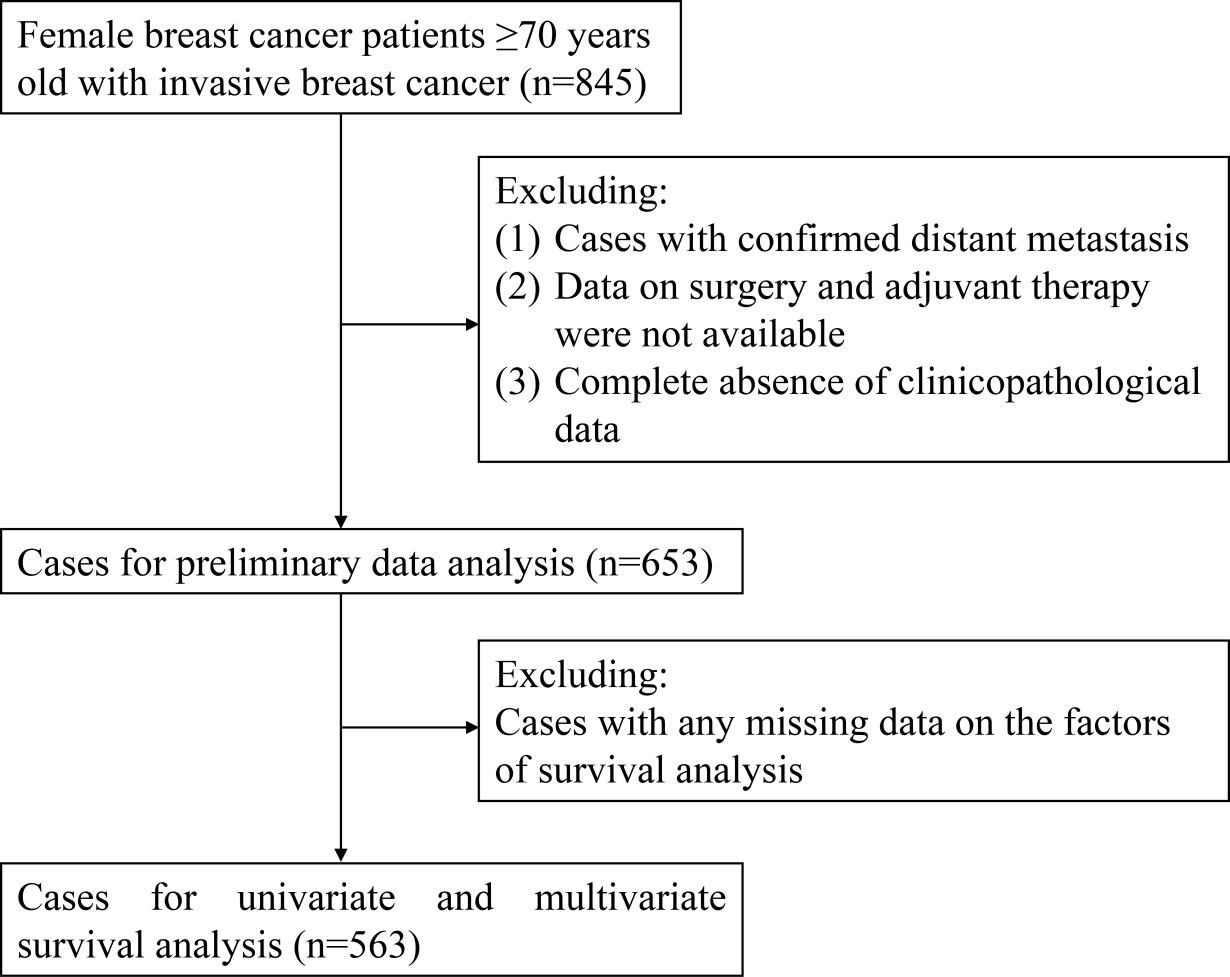
Flow diagram for case inclusion and screening.

From May 2013 to July 2021, a total of 845 patients were roughly included in this study. After screening, a total of 653 patients (mean age, 75.9 years; range, 70–96 years) were enrolled for preliminary data analysis, and finally, 563 patients were selected for survival analysis. The flow diagram for case inclusion and screening is shown in Figure 1. The mean follow-up was 19 months (range, 1–82 months). The clinicopathological characteristics of enrolled cases are presented in Table 1. There was no significant difference in age at diagnosis among the three molecular subtypes (p = 0.071). The majority (68.6%) of patients had HR+/HER2− subtype. Of the enrolled patients, 86.1% were diagnosed with clinical stage I–II. For primary tumor surgery, 91.1% of patients underwent mastectomy, 8.3% underwent breast-conserving surgery (BCS), while 0.6% did not undergo any breast surgery (for unknown reasons). For axillary surgery, 47.5% of patients received only sentinel lymph node biopsy (SLNB), 48.8% axillary lymph node dissection (ALND), and a small percentage (3.7%) did not receive any axillary surgery (for unknown reasons). The overall ALN metastasis rate was 37.1%. Of the patients, 32.6% received chemotherapy, while only 10.9% received radiotherapy. Among HR+/HER2− patients, 75.0% received endocrine therapy.

**Table 1:** Clinicopathological characteristics of enrolled cases according to molecular subtypes.

Factors	Total	HR+/HER2−	HER2+	TNBC	p-value^a^
**Mean age (year)**		76.0	74.9	76.4	0.071
**cN**					<0.001
Negative	411	299	45	67	
Positive	242	149	57	36	
**Clinical stage**					<0.001
I	231	181	19	31	
II	331	226	53	52	
III	91	41	30	20	
**Breast surgery**					0.008
No surgery	4	3	1	0	
BCS	54	46	1	7	
Mastectomy	595	399	100	96	
**Axillary surgery**					0.001
No surgery	24	17	3	4	
SLNB	310	234	31	45	
ALND	319	197	68	54	
**Pathological type**					0.032
IDC	625	424	102	99	
ILC	28	24	0	4	
**Size of invasive carcinoma**					0.025
≤2cm	307	228	37	42	
>2cm	340	215	64	61	
NA	6	5	1	0	
**pN**					0.005
Negative	393	282	45	66	
Positive	242	153	55	34	
NA	18	13	2	3	
**Ki-67**					<0.001
<15%	97	87	2	8	
≥15%	554	361	98	95	
NA	2	0	2	0	
**Chemotherapy**					<0.001
No	440	346	43	51	
Yes	213	102	59	52	
**Radiotherapy**					0.101
No	579	405	84	90	
Yes	71	40	18	13	
NA	3	3	0	0	
**Endocrine therapy**					<0.001
No		85	62		
Yes		336	28		
NA		27	12		

HR, hormone receptor; HER2, human epidermal growth factor receptor 2; TNBC, triple negative breast cancer; cN, clinical status of axillary lymph nodes; BCS, breast-conserving surgery; SLNB, sentinel lymph node biopsy; ALND, axillary lymph node dissection; IDC, invasive ductal carcinoma; ILC, invasive lobular carcinoma; pN, pathological status of axillary lymph nodes; NA, data not available.

^a^ Significance was tested via Fisher’s exact test (except age between two groups was tested via Student’s t-test).

### Axillary surgery might be safely omitted for patients with cN0

According to the axillary surgical protocol, we further divided patients into only SLNB group (SLNB group) and ALND group (including direct ALND and SLNB+ALND). As shown in Table 2, among 240 cN+ cases undergoing axillary surgery, 233 (97.1%) were ultimately identified as pathological ALN metastases (97.3% in HR+/HER2−, 98.1% in HER2+, and 94.4% in TNBC).

**Table 2:** Analysis of pathological axillary lymph node status in various axillary surgeries among cases with clinical lymph node negative (cN0) and clinical lymph node positive (cN+).

Subtype	pN	cN0		cN+	
		SLNB (%)	ALND (%)	SLNB (%)	ALND (%)
**All**	Negative	280 (98.2)	101 (97.1)	1 (4.0)	6 (2.8)
	Positive	5 (1.8)	3 (2.9)	24 (96.0)	209 (97.2)
	Total	285	104	25	215
**HR+/HER2−**	Negative	209 (97.7)	65 (95.6)	1 (5.0)	3 (2.3)
	Positive	5 (2.3)	3 (4.4)	19 (95.0)	126 (97.7)
	Total	214	68	20	129
**HER2+**	Negative	29 (100)	15 (100)	0 (0)	1 (1.9)
	Positive	0 (0)	0 (0)	2 (100)	52 (98.1)
	Total	29	15	2	53
**TNBC**	Negative	42 (100)	21 (100)	0 (0)	2 (6.1)
	Positive	0 (0)	0 (0)	3 (100)	31 (93.9)
	Total	42	21	3	33

HR, hormone receptor; HER2, human epidermal growth factor receptor 2; TNBC, triple negative breast cancer; pN, pathological status of axillary lymph nodes; SLNB, sentinel lymph node biopsy; ALND, axillary lymph node dissection.

ALN metastases were pathologically confirmed (pN+) in only 8 of 389 patients (2.1%) with cN0 (1.8% in SLNB group and 2.9% in ALND group). In HR+/HER2− subgroup, 8 of 282 (2.8%) cN0 cases were ultimately confirmed to be pN+ (Table 2). However, in HER2+ and TNBC subgroup, all cases of cN0 were pN0 eventually (Table 2). Therefore, axillary surgery might be safely omitted for elderly breast cancer patients with cN0.

### Surgical extent of elderly breast cancer was expected to be narrowed down

**Figure 2:**
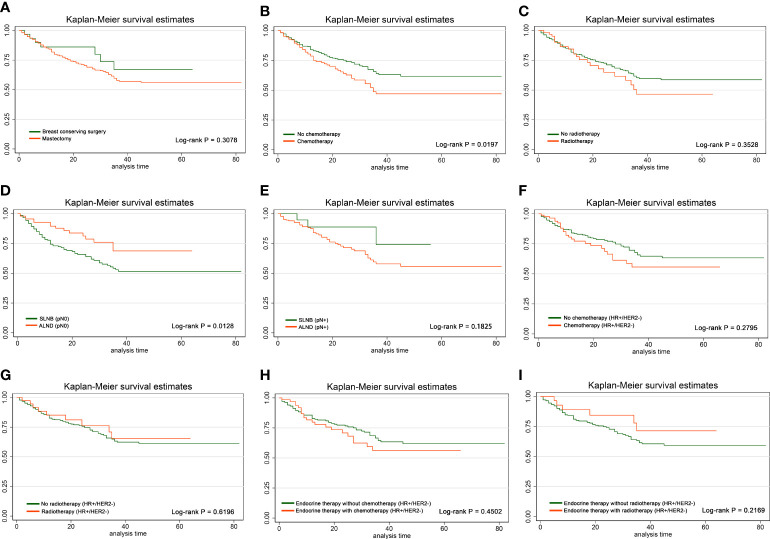
Effects of different local and systemic treatments on RFS. Kaplan–Meier curves and log-rank p-values in the comparison between **(A)** mastectomy group and BCS group, **(B)** chemotherapy group and non-chemotherapy group, **(C)** radiotherapy group and non-radiotherapy group, **(D)** SLNB group and ALND group among pN0 cases, **(E)** SLNB group and ALND group among pN+ cases, **(F)** chemotherapy group and non-chemotherapy group among HR+/HER2− cases, **(G)** radiotherapy group and non-radiotherapy group among HR+/HER2− cases, **(H)** chemotherapy group and non-chemotherapy group among HR+/HER2− cases who have already received endocrine therapy, and **(I)** radiotherapy group and non-radiotherapy group among HR+/HER2− cases who have already received endocrine therapy.

In the entire population, we divided the included cases into mastectomy group and BCS group. After log-rank test, RFS showed no significant difference between mastectomy group and BCS group (p = 0.3078) (Figure 2A), suggesting that some eligible patients were supposed to be exempted from non-essential mastectomy procedures, which could decrease surgical trauma and complications. The clinicopathological characteristics between the two groups are shown in Supplementary Table S1.

Furthermore, we divided the cases into pN0 and pN+ subgroup according to pathological ALN status for stratification analysis of the difference of RFS between the SLNB and ALND group. Consequently, patients in the ALND group represented significantly better RFS than those in the SLNB group among pN0 patients (p = 0.0128), while there was no such effect in pN+ patients (p = 0.1825) (Figures 2D, E). The distribution of clinicopathological features of SLNB and ALND in pN0 patients is further investigated in Table 3. Patients who received ALND were older and had a higher proportion of stage II–III, larger size of invasive carcinoma, and higher Ki-67 expression (all p-values <0.05). They were more likely to undergo mastectomy (p = 0.016). The proportions of chemotherapy and radiotherapy were similar between SLNB and ALND patients, as were pathological types (all p-values >0.05).

**Table 3:** Clinicopathological characteristics of pN0 cases according to axillary surgery.

Factors	SLNB	ALND	P value^a^
**Mean age (range)**	75.3 (70–91)	76.8 (70–92)	0.009
**cN**			0.002
Negative	257 (99.6%)	90 (93.6%)	
Positive	1 (0.4%)	6 (6.4%)	
**Clinical stage**			<0.001
I	159 (61.6%)	37 (38.5%)	
II	99 (38.4%)	56 (58.3%)	
III	0 (0%)	3 (3.1%)	
**Breast surgery**			0.016
BCS	23 (8.9%)	2 (2.1%)	
Mastectomy	235 (91.1%)	94 (97.9%)	
**Pathological type**			0.525
IDC	243 (94.2%)	90 (93.7%)	
ILC	15 (5.8%)	6 (6.3%)	
**Size of invasive carcinoma**			<0.001
≤2cm	160 (62.0%)	39 (40.6%)	
≥2cm	98 (38.0%)	57 (59.4%)	
**Molecular subtype**			0.119
HR+/HER2−	197 (76.4%)	63 (65.6%)	
HER2+	25 (9.7%)	14 (14.6%)	
TNBC	36 (13.9%)	19 (19.8%)	
**Ki-67**			0.046
>15%	54 (20.9%)	12 (12.5%)	
≥15%	204 (79.1%)	84 (87.5%)	
**Chemotherapy**			0.084
No	210 (81.4%)	71 (74.0%)	
Yes	48 (18.6%)	25 (26.0%)	
**Radiotherapy**			0.583
No	249 (96.5%)	93 (96.9%)	
Yes	9 (3.5%)	3 (3.1%)	

SLNB, sentinel lymph node biopsy; ALND, axillary lymph node dissection; cN, clinical ALN status; BCS, breast-conserving surgery; IDC, invasive ductal carcinoma; ILC, invasive lobular carcinoma; HR, hormone receptor; HER2, human epidermal growth factor receptor 2; TNBC, triple negative breast cancer.

^a^Significance was tested via Fisher’s exact test.

To explore why patients in the ALND group had better RFS than those in the SLNB group in pN0 patients, we reviewed the status of ALNs on preoperative imaging in these patients and divided them into cN0 and cN+. The proportion of cN+ in the ALND group was significantly higher than that in the SLNB group (ALND, 6/96 [6.4%]; SLNB, 1/258; [0.4%], p = 0.002) (Table 3), which meant that ALNs of the ALND group were larger in size by imaging and more likely to be misdiagnosed as metastatic. Therefore, in pN0 patients, there was a certain proportion of patients with preoperative false-positive ALN results, and thus, the axilla was overtreated.

### Comprehensive adjuvant therapy regimens had limited impact on RFS especially in HR+/HER2− cases

After log-rank test in the total population, radiotherapy had no significant effect on RFS (p = 0.3528), but patients receiving chemotherapy had worse RFS (p = 0.0197) (Figures 2B, C). In HR+/HER2− population, neither chemotherapy nor radiotherapy affected RFS, and similar conclusion was drawn in HR+/HER2− patients who had already received endocrine therapy (all p-values >0.05) (Figures 2F–I).

Factors that might influence RFS were included in the univariate and multivariate analyses, which was stratified into HR+/HER2−, HER2+, and TNBC subgroups. In the HER2+ and TNBC subgroups, we divided cases into chemotherapy alone group (C) and chemotherapy + radiotherapy group (C+R) according to the adjuvant therapy regimens. There was no significant difference in RFS between C group and C+R group in these two subtypes (Supplementary Table S2). However, sample sizes were limited in these two subgroups, so these results needed further confirmation (HER2+, n = 32; TNBC, n = 39).

Ulteriorly, we assigned HR+/HER2− patients (n = 360) to no adjuvant therapy group (None), endocrine therapy alone group (E), endocrine therapy + chemotherapy group (E+C), and endocrine therapy + chemotherapy + radiotherapy group (E+C+R). In this subgroup, patients who did not receive any adjuvant therapy accounted for 15.7%. The results showed that there was no significant difference in RFS between the four groups in both univariate and multivariate analyses (all p-values >0.05) (Table 4). All these results suggested that the effect of comprehensive adjuvant therapy regimens on RFS was not strong, especially in HR+/HER2− cases.

**Table 4:** Univariate and multivariate analysis of RFS among HR+/HER2− patients.

Factors	Univariate analysis		Multivariate analysis	
	Hazard ratio (95% CI)	p-value	Hazard ratio (95% CI)	p-value
**Age**	0.983 (0.941–1.029)	0.473	1.001 (0.953–1.052)	0.972
**Size of invasive carcinoma**				
≤2cm	Ref.		Ref.	
≥2cm	0.996 (0.647–1.534)	0.987	1.024 (0.651–1.612)	0.917
**Breast surgery**				
BCS	Ref.		Ref.	
Mastectomy	1.028 (0.448–2.359)	0.949	1.019 (0.417–2.490)	0.967
**Axillary surgery**				
SLNB	Ref.		Ref.	
ALND	0.586 (0.373–0.921)	0.021	0.602 (0.350–1.036)	0.067
**pN**				
Negative	Ref.		Ref.	
Positive	0.664 (0.405–1.089)	0.105	0.807 (0.438–1.488)	0.493
**Ki-67**				
>15%	Ref.		Ref.	
≥15%	1.446 (0.784–2.667)	0.238	1.520 (0.808–2.860)	0.195
**Adjuvant therapy**				
None	Ref.		Ref.	
E	1.876 (0.750–4.692)	0.178	1.893 (0.749–4.784)	0.177
E+C	2.325 (0.862–6.273)	0.096	2.501 (0.888–7.049)	0.083
E+C+R	1.531 (0.365–6.418)	0.560	2.002 (0.446–8,983)	0.365

HR, hormone receptor; HER2, human epidermal growth factor receptor 2; BCS, breast-conserving surgery; SLNB, sentinel lymph node biopsy; ALND, axillary lymph node dissection; pN, pathological status of axillary lymph nodes; E, endocrine therapy; C, chemotherapy; R, radiotherapy.

## Discussion

Elderly patients make up a special group of breast cancer patients. The treatment strategy for breast cancer in the elderly is still controversial. Despite receiving the same guideline-concordant treatment, elderly patients still die more prematurely than their younger counterparts (16). For elderly patients with breast cancer, multiple factors should be fully considered, such as biological age, expected remaining life, treatment risk/benefit, and patients’ willingness and tolerance, in order to work out the optimal therapeutic schedule (3). The toxicity of chemotherapeutic drugs (such as hematological disorders, cardiac diseases, and loss of cognitive function) cannot be ignored due to the decline of liver and kidney function caused by physiological senescence (17–20). Likewise, the heart and lung could be damaged by radiotherapy as well (21, 22). Some scholars considered that treatment can be based on standard guidelines for healthy elderly patients (23). Nevertheless, it is important to note that a significant proportion of elderly patients have one or more underlying diseases and may have some other concomitant diseases. Therefore, it is not appropriate to completely refer to younger patients in the treatment of elderly breast cancer (24).

In the past years, there have been multiple research results that support the de-escalation therapy for elderly breast cancer. A study of 57,351 breast cancer patients aged 70 years or older based on SEER database found that the proportion of elderly patients undergoing non-surgical treatment increased with age (25). Endocrine therapy is strongly recommended for elderly patients with HR+ breast cancer because of survival benefit, and radiotherapy could be omitted for early ER+ breast cancer patients whose age ≥70 years undergoing breast lumpectomy and endocrine therapy (14, 26, 27). Additionally, the European Society of Breast Cancer Specialists (EUSOMA) and International Society of Geriatric Oncology (SIOG) also recommended endocrine therapy as the first treatment for elderly breast cancer patients who are not eligible for surgery (28, 29). A multicenter prospective observational study of 3,456 breast cancer patients aged ≥70 years showed that chemotherapy reduced the risk of metastasis but did not significantly improve OS and BCSS. Chemotherapy improved OS and BCSS only in ER− cases (30). In an open-label randomized controlled trial of 275 HER2+ patients aged 70–80 years with breast cancer, trastuzumab monotherapy had a survival loss of <1 month but lower toxicity and higher health-related quality of life compared with trastuzumab plus chemotherapy (15). A prospective, multicenter, observational cohort study of 3,416 patients aged ≥70 years showed that surgery followed by endocrine therapy improved OS compared with endocrine therapy alone, but had no significant effect on BCSS, local recurrence, or distant metastasis in ER+ cases (31). A retrospective study of 3,361 breast cancer patients ≥70 years of age indicated that SLNB and radiotherapy did not improve RFS and DFS in elderly patients with HR+, cN0 breast cancer (32). Besides, there was no significant association between ALND and BCSS in cN0 breast cancer patients aged ≥70 years at 15 years of follow-up, and the addition of radiotherapy had no significant effect on ipsilateral breast cancer recurrence for pT1 patients (33). A meta-analysis published in 2021 suggested that radiotherapy reduced the risk of ipsilateral breast recurrence in elderly patients undergoing BCS but had no significant effect on OS (34). As can be seen from above, these studies mostly focus on one or two points in local and systemic treatment. In our study, stratified by molecular subtypes, we comprehensively and systematically investigated the effects of different treatment strategies on the prognosis of elderly breast cancer patients.

After log-rank test among total population, RFS of the radiotherapy group was similar to that of the non-radiotherapy group, while RFS of the chemotherapy group was significantly worse than that of the non-chemotherapy group (p = 0.0197), which may be due to more aggressive tumor biological behavior in the chemotherapy group. Nonetheless, there was no significant difference in HR+/HER2− or in those who had already received endocrine therapy. Moreover, RFS showed no significant difference between mastectomy and BCS group, SLNB, and ALND. These results suggested that some eligible elderly patients were supposed to be exempted from non-essential mastectomy procedures, which could lessen surgical trauma and complications.

When conducting univariate and multivariate analyses, RFS was not affected by age, invasive cancer size, breast and axillary surgical approach, or pathological ALN status in all three molecular subtypes (Table 4; Supplementary Table S2). In HR+/HER2− patients, RFS in different combinations of adjuvant therapy (no adjuvant therapy, endocrine therapy alone, endocrine therapy + chemotherapy and endocrine therapy + chemotherapy + radiotherapy) were similar. In HER2+ and TNBC patients, there was no significant difference in RFS between chemotherapy alone and chemotherapy + radiotherapy group. However, the number of cases in the HER2+ and TNBC subgroups was small; thus, the conclusion needs further confirmation. These data provided further evidence for the de-escalation of comprehensive therapy particularly in HR+/HER2− elderly breast cancer.

In a systematic review published in *JAMA Surgery*, SLNB was considered as “low-value surgery” for breast cancer patients aged ≥70 years with HR+ and cN0 (35). Our statistics showed that only 2.1% of cN0 patients were ultimately identified as pathological ALN metastases, which confirmed the conclusion of the above review. Such a low pathological ALN positive rate in cN0 patients provided evidence for the exemption of SLNB in a suitable subset of elderly patients. Furthermore, 97.1% of cN+ cases were finally confirmed pathological ALN metastases. Therefore, axillary intervention should be especially carried out with caution in patients with cN+.

Further analysis showed that patients in the ALND group had a significantly better RFS than those in the SLNB group among pN0 patients, while there was no such difference in pN+ cases. In view of this phenomenon, we further divided the patients into cN0 and cN+ by reviewing the status of ALNs on preoperative imaging. In general, cN+ meant that the ALN was larger in size in imaging. We found that the proportion of cN+ in the ALND group was significantly higher than that in the SLNB group. A study published in 2020 by Chen et al. compared the outcomes of patients with cN0 and cN1 in preoperative assessment among 692 pN0 cases, and BCSS of cN1 patients was unexpectedly significantly longer than cN0 ones in TNBC. Using RNA sequencing, they found that enlarged ALNs in clinical imaging had higher expression of immune-related genes, such as *IL21*, *CCL17*, *AOC1*, *APOC2*, and *NCCRP1*, and more abundant immune cells, such as dendritic cells, CD4+ T cells, and CD8+ T cells (36). Increased anti-tumor immunity improved survival, which partly explained our findings. False positive results of preoperative axillary lymph node assessment in patients with pN0 suggested that the axilla might be overtreated in elderly breast cancer patients. Hence, more accurate preoperative assessment of ALN, such as ultrasound-guided lymph node biopsy, is particularly crucial in elderly patients with cN+. On the other hand, it also suggested that the role of immunotherapy in elderly breast cancer deserves further study.

There were some limitations in our study. First, retrospective design inevitably had biases such as recalling bias, selection bias, withdraw bias, and confounding bias. The cases included in this study were from different medical teams at the same institution, which would lead to inconsistency in treatment preferences. Second, OS and BCSS were hard to analyze in our study because of the low incidence of death events in follow-up. Third, due to the long time span of this study and the lack of partial data on molecular targeted therapy, we did not analyze the impact of anti-HER-2 therapy on prognosis of elderly breast cancer patients. Importantly, it is worth mentioning that the sample size of HER2+ and TNBC subtypes in this study was small.

In conclusion, it is feasible to reduce some unnecessary local or systemic treatments for breast cancer in Chinese women aged 70 years or older, especially in HR+/HER2− subtype. Endocrine therapy is still recommended for HR+ patients, which has been confirmed in several large studies. When making treatment plans, surgeons should take multiple patient-related factors into account, such as physical condition and subjective willingness. Rational de-escalation therapy may improve the quality of life of elderly patients without influencing the prognosis. In an era when the comprehensive treatment of breast cancer has been momentously improved, more high-quality, large-sample prospective studies are urgently needed to ulteriorly demonstrate the feasibility and safety of de-escalation therapy for elderly breast cancer.

## Data availability statement

The raw data supporting the conclusions of this article will be made available by the authors, without undue reservation.

## Ethics statement

Ethical review and approval was not required for the study on human participants in accordance with the local legislation and institutional requirements. Written informed consent from the patients/participants or patients/participants' legal guardian/next of kin was not required to participate in this study in accordance with the national legislation and the institutional requirements.

## Author contributions

Study conception and design were carried out by SW, WZ, and HP. Follow-up and data collection were performed by JW, HF, ZZ, YJ, XS, WX, and XT. Data statistics and analysis were completed by JW and HF. The first draft of the manuscript was written by JW and HF. SW, WZ, YJ, and ZZ commented on previous versions of the manuscript. All authors read and approved the final manuscript.

## Funding

This study was supported by the Natural Science Foundation of China (81771953 and 82172613), the Natural Science Foundation of Jiangsu Province (BK20180108), and a project funded by the Priority Academic Program Development of Jiangsu Higher Education Institutions.

## Conflict of interest

The authors declare that the research was conducted in the absence of any commercial or financial relationships that could be construed as a potential conflict of interest.

## Publisher’s note

All claims expressed in this article are solely those of the authors and do not necessarily represent those of their affiliated organizations, or those of the publisher, the editors and the reviewers. Any product that may be evaluated in this article, or claim that may be made by its manufacturer, is not guaranteed or endorsed by the publisher.
