# Supplementary material for: Local and systemic therapy may be safely de-escalated in elderly breast cancer patients in China: A retrospective cohort study
Source: Front Oncol. 2022 Jul 28;12:958116. doi: 10.3389/fonc.2022.958116 (PMC9371841; doi:10.3389/fonc.2022.958116)
Supplement: Supplementary file 1 [file Table_1.docx]

**Supplementary Table 1** Clinicopathological characteristics of enrolled cases according to breast surgery

| **Factors** | **BCS** | **Mastectomy** | **P value^a^** |
| --- | --- | --- | --- |
| **Mean age (range)** | 78.0 (70-92) | 75.7 (70-96) | 0.001 |
| **cN** |  |  | 0.018 |
| Negative | 42 (77.8%) | 365 (61.3%) |  |
| Positive | 12 (22.2%) | 230 (38.7%) |  |
| **Clinical stage** |  |  | ＜0.001 |
| Ⅰ | 31 (57.4%) | 200 (33.6%) |  |
| Ⅱ | 22 (40.7%) | 306 (51.4%) |  |
| Ⅲ | 1 (1.9%) | 89 (15.0%) |  |
| **Pathological type** |  |  | 1.000 |
| IDC | 52 (96.3%) | 569 (95.6%) |  |
| ILC | 2 (3.7%) | 26 (4.4%) |  |
| **Size of invasive carcinoma** |  |  | ＜0.001 |
| ≤2cm | 39 (72.2%) | 268 (45.0%) |  |
| ＞2cm | 15 (27.8%) | 325 (54.6%) |  |
| NA | 0 (0) | 2 (3.4%) |  |
| **Molecular subtype** |  |  | 0.002 |
| HR+/HER2- | 46 (85.2%) | 399 (67.1%) |  |
| HER2+ | 1 (1.8%) | 100 (16.8%) |  |
| TNBC | 7 (13.0%) | 96 (16.1%) |  |
| **Ki-67** |  |  | 0.520 |
| ＜15% | 10 (18.5%) | 87 (14.6%) |  |
| ≥15% | 44 (81.5%) | 506 (85.0%) |  |
| NA | 0 (0) | 2 (3.4) |  |

*BCS* breast-conserving surgery, *cN* clinical ALN status, *IDC* invasive ductal carcinoma, *ILC* invasive lobular carcinoma, *HR* hormone receptor, *HER2* human epidermal growth factor receptor 2, *TNBC* triple negative breast cancer

^a^ Significance was tested via Fisher's exact test (except age between two groups was tested via student t test).
