# Supplementary material for: Local and systemic therapy may be safely de-escalated in elderly breast cancer patients in China: A retrospective cohort study
Source: Front Oncol. 2022 Jul 28;12:958116. doi: 10.3389/fonc.2022.958116 (PMC9371841; doi:10.3389/fonc.2022.958116)
Supplement: Supplementary file 2 [file Table_2.docx]

**Supplementary Table 2** Univariate and multivariate analysis of RFS among HER2+ and TNBC patients

| **Subtypes** | **HER2+** | | | | **TNBC** | | | |
| --- | --- | --- | --- | --- | --- | --- | --- | --- |
| **Factors** | **Univariate analysis** | | **Multivariate analysis** | | **Univariate analysis** | | **Multivariate analysis** | |
|  | **Hazard ratio (95% CI)** | **P value** | **Hazard ratio (95% CI)** | **P value** | **Hazard ratio (95% CI)** | **P value** | **Hazard ratio (95% CI)** | **P value** |
| **Age** | 1.063 (0.861-1.312) | 0.569 | 1.238 (0.858-1.786) | 0.254 | 0.983 (0.882-1.094) | 0.751 | 0.983 (0.863-1.119) | 0.791 |
| **Size of invasive carcinoma** |  |  |  |  |  |  |  |  |
| ≤2cm | Ref. |  | Ref. |  | Ref. |  | Ref. |  |
| ＞2cm | 1.280 (0.318-5.143) | 0.728 | 0.682 (0.106-4.378) | 0.687 | 0.716 (0.258-1.986) | 0.521 | 0.698 (0.216-2.259) | 0.549 |
| **Breast surgery** |  |  |  |  |  |  |  |  |
| BCS | Ref. |  | Ref. |  | Ref. |  | Ref. |  |
| Mastectomy | NA | NA | NA | NA | NA | NA | NA | NA |
| **Axillary surgery** |  |  |  |  |  |  |  |  |
| SLNB | Ref. |  | Ref. |  | Ref. |  | Ref. |  |
| ALND | 0.644 (0.129-3.209) | 0.591 | 0.125 (0.003-5.302) | 0.277 | 1.002 (0.340-2.953) | 0.997 | 0.963 (0.236-3.936) | 0.958 |
| **pN** |  |  |  |  |  |  |  |  |
| Negative | Ref. |  | Ref. |  | Ref. |  | Ref. |  |
| Positive | 0.775 (0.192-3.127) | 0.721 | 1.705 (0.088-33.098) | 0.724 | 1.221 (0.439-3.395) | 0.701 | 1.125 (0.172-7.358 | 0.902 |
| **Ki-67** |  |  |  |  |  |  |  |  |
| ＜15% | Ref. |  | Ref. |  | Ref. |  | Ref. |  |
| ≥15% | 0.066 (0.006-0.727) | 0.026 | 0.031 (0.002-0.593) | 0.021 | 0.121 (0.013-1.082) | 0.059 | 0.119 (0.011-1.317) | 0.083 |
| **Adjuvant therapy** |  |  |  |  |  |  |  |  |
| C | Ref. |  | Ref. |  | Ref. |  | Ref. |  |
| C+R | 0.896 (0.222-3.613) | 0.878 | 1.793 (0.277-11.597) | 0.540 | 1.654 (0.609-4.493) | 0.323 | 1.809 (0.402-8.145) | 0.440 |

HER2, human epidermal growth factor receptor 2; TNBC, triple negative breast cancer; BCS, breast-conserving surgery; SLNB, sentinel lymph node biopsy; ALND, axillary lymph node dissection; pN, pathological status of axillary lymph nodes; C, chemotherapy; R, Radiotherapy; NA, data not available.
